# Revealing composition and structure dependent deep-level defect in antimony trisulfide photovoltaics

**DOI:** 10.1038/s41467-021-23592-0

**Published:** 2021-05-31

**Authors:** Weitao Lian, Chenhui Jiang, Yiwei Yin, Rongfeng Tang, Gang Li, Lijian Zhang, Bo Che, Tao Chen

**Affiliations:** 1 Hefei National Laboratory for Physical Sciences at Microscale, CAS Key Laboratory of Materials for Energy Conversion, Department of Materials Science and Engineering, School of Chemistry and Materials Science, University of Science and Technology of China, Hefei, Anhui P. R. China; 2 Institute of Energy, Hefei Comprehensive National Science Center, Hefei, China

**Keywords:** Solar cells, Solar cells, Structural properties

## Abstract

Antimony trisulfide (Sb_2_S_3_) is a kind of emerging light-harvesting material with excellent stability and abundant elemental storage. Due to the quasi-one-dimensional symmetry, theoretical investigations have pointed out that there exist complicated defect properties. However, there is no experimental verification on the defect property. Here, we conduct optical deep-level transient spectroscopy to investigate defect properties in Sb_2_S_3_ and show that there are maximum three kinds of deep-level defects observed, depending on the composition of Sb_2_S_3_. We also find that the Sb-interstitial (Sb_i_) defect does not show critical influence on the carrier lifetime, indicating the high tolerance of the one-dimensional crystal structure where the space of (Sb_4_S_6_)_n_ ribbons is able to accommodate impurities to certain extent. This study provides basic understanding on the defect properties of quasi-one-dimensional materials and a guidance for the efficiency improvement of Sb_2_S_3_ solar cells.

## Abstract (web summary)

Antimony trisulfide emerges as a suitable candidate for light-harvesting due to its good stability and abundance, yet the defect properties are not well-understood. Here, by means of deep-level transient spectroscopy, Lian et al. find that there are three kinds of deep-level defects depending on the chemical composition.

## Introduction

Point defect engineering of semiconducting materials is essential for photovoltaic devices, comprehensive understanding of the defect formation mechanism and function enables achieving high-efficiency solar energy conversion. In specific, the point defect (intrinsic or extrinsic defect) determines the Fermi level (*E*_F_), free carrier concentration, and conductivity type (n or p) of semiconductors. Shallow-level defects with thermal energy of about *k*_B_*T* (where *k*_B_ is Boltzmann constant, *T* is temperature) from conduction band minimum (CBM) or valence band maximum (VBM) play the major role to tune the carrier concentrations and conductivity type. In contrast, deep-level defect whose activation energy is much higher than *k*_B_*T* from CBM or VBM is detrimental to photogenerated carrier lifetime and transport (carrier mobility and diffusion length)^1^. These defects result in the trap-assisted Shockley–Read–Hall^2,3^ (SRH) recombination (dominant non-radiation recombination) in solar cells, which is the primary cause of open-circuit voltage (*V*_OC_) loss.

**Fig. 1:**
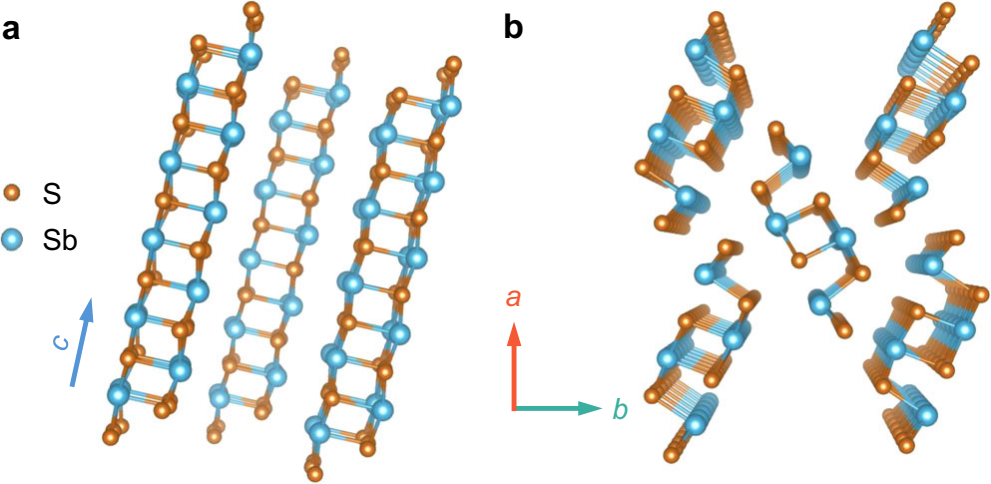
Schematic diagram of quasi-1-dimensional structural Sb_2_S_3_. Sideview (**a**) and aeroview (**b**) of [Sb_4_S_6_] ribbons along *c* axis.

Recently, binary antimony chalcogenides Sb_2_S(e)_3_ (including Sb_2_S_3_, Sb_2_Se_3_, Sb_2_(S, Se)_3_ alloy) have drawn increasing attention in solar cell applications, for their large visible light absorption coefficient (>10^5^ cm^−1^), earth-abundant compositional elements, and tunable band gap in 1.1–1.7 eV. The efficiency breakthrough towards 10% in alloy-type antimony selenosulfide stimulates new interests in the development of this class of materials^4,5^. In particular, Sb_2_S_3_ with band gap of ~1.7 eV can be perfectly applied as top cell material for the construction of tandem solar cells. Different from previous photovoltaic materials such as silicon, Cu(In, Ga)Se_2_, CdTe, and organic–inorganic hybrid perovskite, the Sb_2_S_3_ displays quasi-one-dimensional structure composed of [Sb_4_S_6_]_n_ ribbons and could generate benign grain boundaries without dangling bonds along *c* axis (Fig. 1)^6,7^. In practical, to transfer this distinctive structural advantage into efficient carrier transport and final power conversion efficiency (PCE), one must understand and control the defect properties, in particular the deep-level defects. This requirement has spurred intense interests in exploring the defect properties of such materials, especially from theoretical perspective, which revealed complicated defect characteristics although it has only two kinds of elements^8–11^. However, to date there is a lack of experimental verification of the defect properties associated with the structure and compositions, which induces ambiguity in the fabrication of high-quality Sb_2_S_3_ films for efficient solar cells.

Herein, we experimentally uncover the defect characteristics by using deep-level transient spectroscopy (DLTS) and identify the details of the defect. We examine both Sb-rich and S-rich Sb_2_S_3_ films for a comparative study which are prepared by thermal evaporation approach. To make clear conclusions, we carefully analyze the crystallinity, electronic structure, and chemical composition (impurity) of the as-obtained films. In contrast to the theoretical study where complicated defect proposed in the Sb_2_S_3_^8–11^, our experimental investigation shows only a few types of defect, and the defect type and concentration are sensitively dependent on the anion/cation ratio.

## Results

### Structure and composition characterization of Sb_2_S_3_ films

**Fig. 2:**
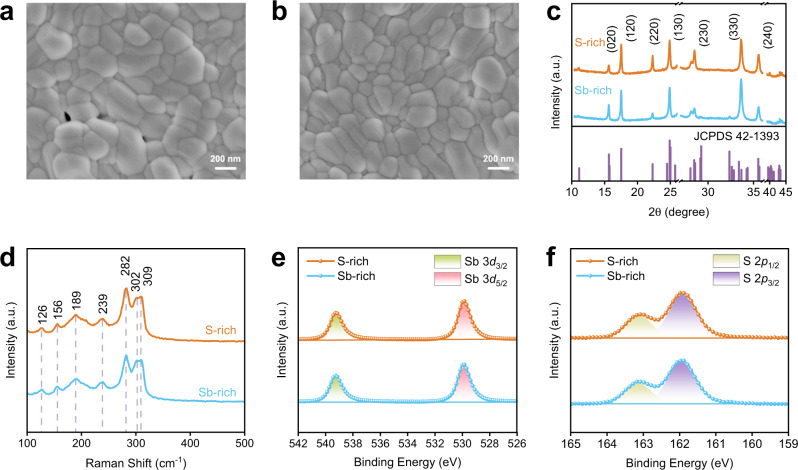
Characterizations of Sb_2_S_3_ films. **a**, **b** Surface SEM images of Sb-rich and S-rich Sb_2_S_3_ films. **c**, **d** XRD patterns and Raman spectroscopy of Sb-rich and S-rich Sb_2_S_3_ films. **e**, **f** Sb 3*d* and S 2*p* XPS spectra of Sb-rich and S-rich Sb_2_S_3_.

Due to the high saturated vapor pressure of sulfur (50 mmHg at 300 °C)^12^, the sulfur loss is inevitable and results in Sb-rich Sb_2_S_3_ films during the normal thermal evaporation deposition. To obtain S-rich Sb_2_S_3_ film, we apply a co-evaporation equipment for the film fabrication, in which S powders are co-evaporated with Sb_2_S_3_ for the generation of S-rich Sb_2_S_3_ films. The synthesis details are provided in the Methods section. The as-obtained films at Sb-rich and S-rich conditions display similar morphology (Fig. 2a, b and Supplementary Fig. 1), featured as compact with clear grain boundaries. X-ray diffraction (XRD) is then applied to characterize the crystallinity (Fig. 2c). Both of the films display diffractions at 15.6, 17.5, 24.9, 33.6, and 35.6°, which are well assigned as (020), (120), (130), (330), and (240) crystal planes of orthorhombic Sb_2_S_3_ (JCPDS No. 42-1393, *Pbnm*)^6^. According to the energy dispersive X-ray spectroscopy (EDS), the atomic ratio of S/Sb in Sb-rich and S-rich Sb_2_S_3_ films are calculated to be 1.28 to 1.55 (Supplementary Table 1), respectively.

Impurity crucially influences the defect properties in a semiconductor. In this case, we carry out Raman scattering and X-ray photoelectron spectroscopy (XPS) characterizations to carefully examine the bulk and surface chemical compositions. Raman spectra show typical asymmetric and symmetric bending vibration of S–Sb–S at 189 and 239 cm^−1^ (Fig. 2d), respectively. The asymmetric and symmetric stretching vibration of Sb–S appear at 282 and 309 cm^−1^. Furthermore, Raman shift at 126 and 156 cm^−1^ correspond to crystalline Sb_2_S_3_ phase^13^. In addition, Raman shift at 302 cm^−1^ is ascribed to the phonon scattering from CdS substrate^14^.

XPS spectra (Fig. 2e, f) display typical binding energy of 539.2 eV and 529.8 eV which are assigned to Sb 3*d*_3/2_ and 3*d*_5/2_ of Sb_2_S_3_, respectively. Notably, there is no SbO_x_ detected in both films, which is usually generated as impurity in the synthesis of Sb_2_S_3_ and displays deep defect characteristics^15^. In addition, the binding energy of 163.1 eV and 161.9 eV are ascribed to the S 2*p*_1/2_ and 2*p*_3/2_ of Sb_2_S_3_^6,15,16^. In any case, from both Raman scattering and XPS analysis, both Sb-rich and S-rich Sb_2_S_3_ films prepared via thermal evaporation are free of impurities, which thus provides excellent platform for the investigations of their defect properties.

### Photovoltaic performance of devices

**Fig. 3:**
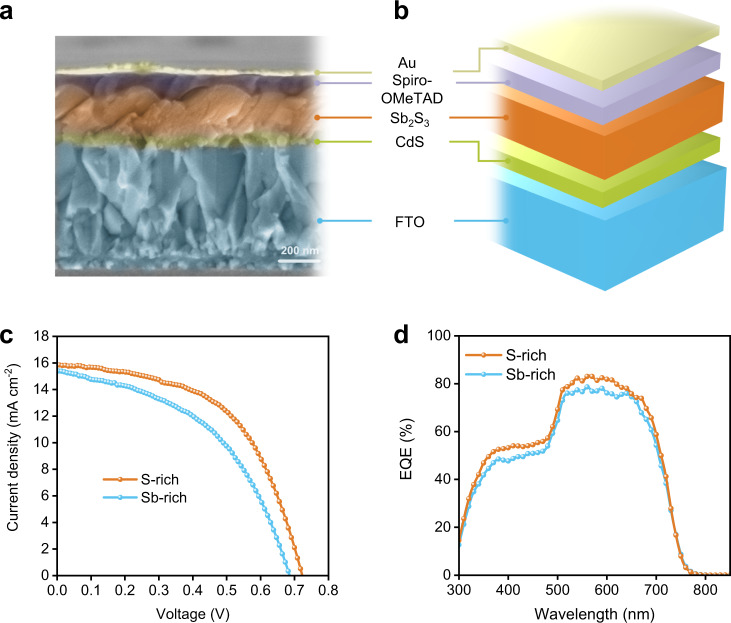
Device structure and photovoltaic performance. **a**, **b** Cross-sectional SEM image and corresponding device configuration of Sb_2_S_3_ solar cells. **c** *J–V* characteristics of the optimal Sb-rich and S-rich Sb_2_S_3_ devices under standard one Sun illumination. **d** EQE spectra of the optimized Sb-rich and S-rich Sb_2_S_3_-based devices.

To test the optoelectronic quality of the as-synthesized film at device level, we examine the solar cell efficiency by using CdS and Spiro-OMeTAD as electron and hole transport layers (ETL & HTL), respectively (Fig. 3a, b). The current density–voltage (*J–V*) curves are obtained under standard AM 1.5 G illumination (Fig. 3c). The optimized Sb-rich Sb_2_S_3_ film-based solar cell delivers PCE of 5.0%, with open-circuit voltage (*V*_OC_), short-circuit current density (*J*_SC_), and fill factor (*FF*) of 0.68 V, 15.4 mA cm^−2^, and 47.6%, respectively. However, the optimized S-rich Sb_2_S_3_ film-based device generates a PCE of 6.2% (*V*_OC_ of 0.72 V, *J*_SC_ of 15.9 mA cm^−2^, and *FF* 54.3%) with a net increase by 1.2% compared to the Sb-rich Sb_2_S_3_-based device. This PCE represents the highest value in thermal evaporation-derived Sb_2_S_3_ solar cells^17,18^. The statistical parameters (*V*_OC_, *J*_SC_, *FF*, and PCE) of each type of devices show narrow distributions (Supplementary Fig. 2a–e), confirming the reliability of the film and device fabrication. Moreover, the external quantum efficiency (EQE) spectra (Fig. 3d and Supplementary Fig. 2f) display excellent photocurrent generation efficiency in the 500–700 nm, a decrease in the short wavelength is attributed to the light absorption loss induced by CdS since its band gap is 2.4 eV. On the ground of both *J–V* and EQE analysis, the as-prepared S-rich Sb_2_S_3_ films display high photovoltaic quality.

### Bulk deep-level defects analysis

We perform DLTS to gain insights into the defects in Sb_2_S_3_. In this characterization, there is a typical concern on the traditional electric DLTS regarding the distortion of minority-carrier trap detection^19^. However, attaching optical pulse to DLTS is recognized as an effective means to improve the sensitivity and authenticity of minority-carrier traps detection^20,21^. Herein, we adopt electric and optical double pulse mode, and both pulses are exerted simultaneously and share the same pulse width. In order to avoid the interference of ETL and HTL, a 635-nm wavelength red laser is selected as the source of optical pulse. Moreover, the reverse bias is set at −0.3 V, while various pulse voltages (0.1–0.5 V) are applied to avoid the fault peaks caused by the capacitance bridge recovery delay at inappropriate pulse voltage sometimes. Meanwhile, the defects at various depths can also be probed by varying the pulse voltage.

**Fig. 4:**
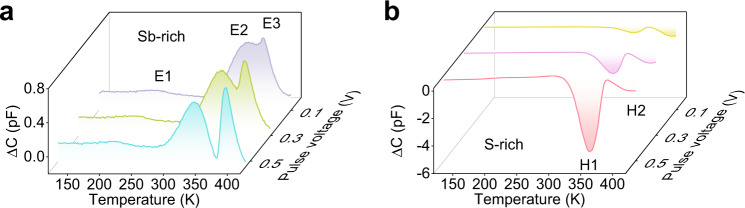
Deep-level defects characterization. DLTS signals of Sb-rich (**a**) and S-rich Sb_2_S_3_ films (**b**) at pulse voltage ranging from 0.1 to 0.5 V, synergized with an identical pulse-width optical pulse. Δ*C* is the variation of capcitance.

**Fig. 5:**
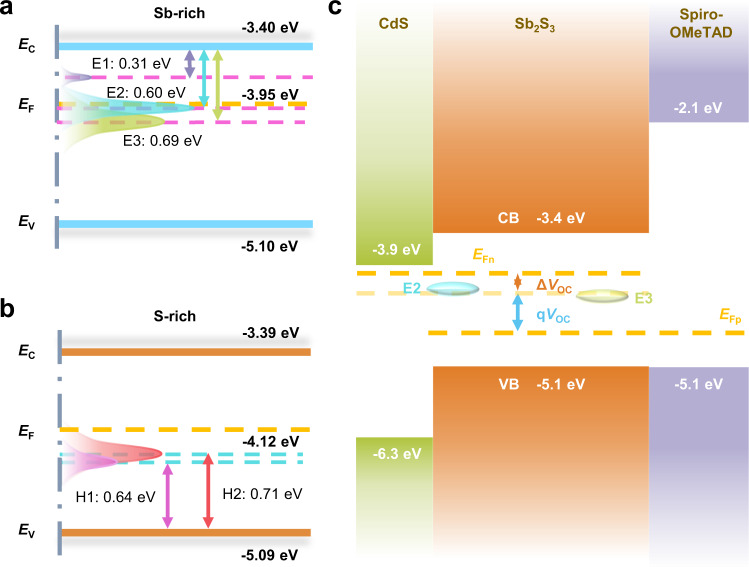
Schematic of band structure and heterojunction. **a**, **b** Conduction band (*E*_C_), valence band (*E*_V_), Fermi level (*E*_F_), and trap energy level (*E*_T_) for Sb-rich and S-rich Sb_2_S_3_ films. **c** Schematic diagram of *V*_OC_ derived from split of electron and hole quasi Fermi level (*E*_Fn_ and *E*_Fp_).

Figure 4a, b shows the DLTS spectra of Sb-rich and S-rich Sb_2_S_3_ devices at different pulse voltages. The positive peaks in the DLTS spectra represent majority-carrier traps, while the negative peaks indicate minority-carrier traps^19^. Regarding n-type Sb_2_S_3_ (Supplementary note 1), the majority and minority-carrier traps are corresponding to electron and hole traps, respectively. Eventually, the statistical defects information of the two kinds of devices can be calculated and the results are summarized in Table 1, where *E*_T_, *σ*, *N*_T_, *τ*, and *N*_S_ are trap energy level, capture cross section, trap density, carrier lifetime, and shallow donor concentration (Supplementary note 2), respectively. It turns out that the Sb-rich film displays three electron traps, E1, E2, and E3 (donor defects), with the energy level of 0.31, 0.60, and 0.69 eV below the CBM (Figs. 4a and 5a). In contrast, the S-rich film exhibits two hole traps which are denoted as H1 and H2 (acceptor defects) with energy levels of 0.64 and 0.71 eV above the VBM (Figs. 4b and 5b). This characterization suggests clearly the composition-depended defect properties of Sb_2_S_3_ films.

**Table 1:** Deep-level defect parameters (trap type, trap energy level (*E*_T_), capture cross section (*σ*), trap density (*N*_T_), carrier lifetime (*τ*), shallow donor concentration (*N*_S_)) of Sb-rich and S-rich Sb_2_S_3_ films.

Sample	Trap	*E*_T_ (eV)	*σ* (cm^2^)	*N*_T_ (cm^-3^)	*τ* (ns)	*N*_S_ (cm^−3^)
Sb-rich	E1	*E*_C_−0.31 ± 0.02	(0.54–8.13) × 10^−17^	(3.75–5.63) × 10^14^	2.18 × 10^3^	7.71 × 10^16^
	E2	*E*_C_−0.60 ± 0.02	(0.26–4.68) × 10^−16^	(1.57–3.31) × 10^15^	6.46 × 10^1^	
	E3	*E*_C_−0.69 ± 0.02	(0.11–1.75) × 10^−15^	(1.38–2.01) × 10^15^	2.84 × 10^1^	
S-rich	H1	*E*_V_ + 0.64 ± 0.01	(0.46–1.31) × 10^−15^	(0.45–1.58) × 10^15^	4.83 × 10^1^	5.13 × 10^16^
	H2	*E*_V_ + 0.71 ± 0.02	(0.49–1.17) × 10^−16^	(6.71–8.57) × 10^14^	9.97 × 10^2^

According to the theoretical calculations^8,9^, the traps E1, E2, E3, H1, and H2 can be well assigned to Sb-interstitial (Sb_i_), S-vacancy (V_S_), Sb_S_ antisite, Sb-vacancy (V_Sb_), and S_Sb_ antisite defects, respectively. In the first place, the S deficit in Sb-rich Sb_2_S_3_ results in the increase of V_S_. Excess Sb will then preferentially fill the V_S_, rather than enter into the interstitial site, since the formation energy of Sb_S_ is essentially lower than that of Sb_i_. Therefore, the defects V_S_ and Sb_S_ are predominantly appeared in Sb-rich Sb_2_S_3_. However, in S-rich Sb_2_S_3_, S atoms enter lattice to fill the V_S_ initially, and the formations of Sb_S_ and Sb_i_ defects are suppressed due to their increased formation energy in S-rich condition. Afterwards, owing to reduced formation energy, the S-rich condition induces the formation of large amount of V_Sb_. Furthermore, some S atoms may even occupy the V_Sb_ to form the S_Sb_ antisite in order to maintain the structural stability.

Notably, there are only maximum three types of defects detected by DLTS in each Sb_2_S_3_ film, much less than those predicted in the theoretical calculations^8^. Fundamentally, the difference between theoretical calculations and experimental measurement can be ascribed to the following two aspects. Firstly, theoretical calculations take all possible defect types into consideration, whereas some of the defects with high formation energy are instable and thus cannot be identified in the measurement. Secondly, DLTS is a technique to detect defects by injecting carriers into depletion region to fill the traps in bulk semiconductors^19^. Accordingly, it is an effective means to probe the deep-level defects in depletion region, especial for which cannot be activated or ionized at room temperature, whereas it is not sensitive to shallow-level defects or defects at interface. These two features result in less defect detected when compared with the theoretical study.

In line with the energy level alignment (Fig. 5a, b), these are all deep-level defects whose energy level is more than 0.3 eV far from CBM or VBM. Therefore, they act as carrier traps or recombination sites due to their high ionization energy. Specially, the trap E2, E3, H1, and H2 feature on deeper energy level (close to intrinsic *E*_F_), large capture cross section and high trap density (Table 1) which accords with basic characteristics of trap-assisted SRH recombination center. Thus, they behave as serious recombination centers to hinder carrier transport and shorten the carrier lifetime.

Although the above Raman and XPS characterizations do not show impurities such as antimony oxides in the as-prepared Sb_2_S_3_ films, we could not exclude the possibility of tiny amount (beyond detection limit) of oxygen doping affecting the defect properties. In this case, we tailor the composition via post-treatment of as-prepared Sb_2_S_3_ films with an O_2_/Ar mixed atmosphere at 200 °C for 20–60 min to intentionally introduce oxygen doping (experimental details are provided in the Supplementary note 3). Both the secondary ion mass spectroscopy (SIMS) and XPS spectra (Supplementary Fig. 5) indicate clearly that there is no oxygen existed in as-prepared Sb_2_S_3_ films. After the annealing at 200 °C in O_2_/Ar, the concentration of oxygen is gradually increased from 20 to 60 min (Supplementary Fig. 5c).

We then carry out DLTS analysis. It turns out that oxygen generates remarkable impact on defect properties of Sb_2_S_3_ films (Supplementary Fig. 6, Supplementary Tables 4 and 5). In Sb-rich Sb_2_S_3_, the previous three electron traps (E1, E2, E3) are no longer detected, along with the appearance of two hole traps (H1 and H3) after O_2_ treatment at 200 °C for 20 min, and even only one hole trap (H3) is observed when the O_2_ post-treatment time is prolonged to 40 min. In S-rich Sb_2_S_3_, the previous hole trap H1 is also substituted with another hole trap H3 after O_2_ post-treatment, we attribute the trap H3 to O_Sb_ according to defect energy level calculation^8^. Furthermore, the H2 is also passivated when the post-treatment in O_2_ was increased to 60 min. On the one hand, we propose that oxygen plays a similar role as sulfur during the processing, which is reasonable since oxygen and sulfur are at the same group in the periodic table. On the other hand, oxygen is more reactive than that of sulfur, it could thus passivate several kinds of defects such as V_S_, Sb_S_, and Sb_i_ caused by S deficit, and excessive oxygen would most likely result in the formation of O_Sb_ defects^8^. It is therefore rational that there is finally only one kind of defect like O_Sb_ in the long time (e.g., 60 min) oxygen-treated Sb_2_S_3_ films no matter whether the original Sb_2_S_3_ is Sb-rich or S-rich (Supplementary Fig. 6).

According to the above discussion, we can conclude that certain amount of oxygen could induce distinct defect property, particularly in the defect types. Considering that there is no oxygen impurities detected in the as-prepared Sb_2_S_3_ films (or negligible amount of oxygen that is beyond detection limit), the defect properties of as-prepared Sb_2_S_3_ film in absence of oxygen post-treatment should not be influenced by the possible impurities.

### Carrier dynamic analysis

**Fig. 6:**
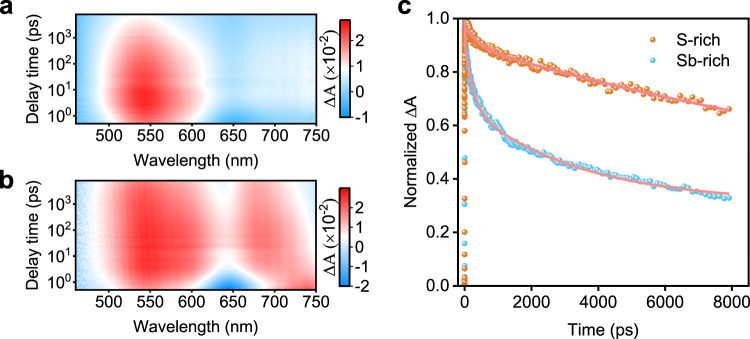
Transient absorption spectroscopy (TAS) study of Sb_2_S_3_ films. **a**, **b** 2D color images of TAS of Sb-rich and S-rich Sb_2_S_3_ films on glass substrates. **c** Transient kinetic decay (scatter) and fittings according to biexponential decay function (solid lines) monitored at 545 nm of Sb-rich and S-rich films. Δ*A* is defined as variation of absorption.

To study the charge transport kinetics, we perform transient absorption spectroscopy (TAS) measurement of the Sb-rich and S-rich Sb_2_S_3_ films deposited on soda-lime glass. We apply glass/Sb_2_S_3_ films without any electron or hole extraction layer as sample for this study, which can reflect unambiguously that the exciton relaxes to ground state through charge recombination in films. The TAS is tracked at a time window of 5−5000 ps using 400 nm pulse laser excitation for Sb-rich and S-rich Sb_2_S_3_ films (Fig. 6a, b, Supplementary Fig. 7). Strikingly, there appear two photo-induced absorption peaks near 545 and 690 nm, both of which are assigned to a single broad feature of trapped carrier in Sb_2_S_3_. Especially, the peak at 690 nm is associated with the formation of sulfide radical^22,23^. Thus, the enhanced absorption at 690 nm for S-rich Sb_2_S_3_ could be attributed to abundant sulfide radical. Accordingly, the decay characteristics at 545 nm are fitted by biexponential model to study carrier decay kinetics (Fig. 6c and Supplementary Table 2). It is observed that the S-rich Sb_2_S_3_ exhibits much longer carrier lifetime (18.7 ns) than Sb-rich Sb_2_S_3_ (3.8 ns), the reduced defect type and concentration contribute to the prolonged lifetime.

To gain further insight into the specific influence of each defect on the carrier dynamics, we extract the limited carrier lifetime associated with specific defect by trap-assisted SRH recombination according to Eq. 1.

1 $$\tau ={\left({\upsilon }_{{\rm{th}}}\sigma {N}_{{\rm{T}}}\right)}^{-1}.$$

where the *υ*_th_ is thermal velocity for electron (hole) that is about 10^7^ cm s^−1^ in bulk semiconductors at room temperature^2,3,24^. Both *σ* and *N*_T_ can be obtained from DLTS. Consequently, the defect with large capture cross section, high density and deep energy level are much more detrimental to carrier lifetime.

In Sb-rich Sb_2_S_3_ film, the carrier lifetime is limited by trap E2 (V_S_) and E3 (Sb_S_) in Sb-rich Sb_2_S_3_ film which are estimated to be 64.6 and 28.4 ns (Table 1), respectively. The other defect E1 (Sb_i_) present carrier lifetime of 2180 ns, much longer than those of E2 and E3. This result should be associated with the crystal structure where the Sb atoms entering into the space between (Sb_4_S_6_)_n_ ribbons is less detrimental to the carrier transport dynamics. In S-rich Sb_2_S_3_ film, the trap H1 (V_Sb_) sets the limited lifetime of 48.3 ns, while the lifetime limited by trap H2 (S_Sb_) is 997 ns. Defect S_Sb_ shows less detrimental to carrier lifetime since the lower concentration due to higher formation energy^9^, even though it manifests deeper energy level than V_Sb_.

We further compare the shortest carrier lifetime limited by E3 and H1, we suppose that this prolonged carrier lifetime is attributed to reduced capture cross section and trap density of H1. In fact, the recombination form in real semiconductors is mainly multi-level recombination^24^. That is to say, the carriers leap among multiple trap level and get recombination ultimately. Therefore, the decreased defect numbers in S-rich Sb_2_S_3_ also contribute the longer carrier lifetime. It is worth noting that the calculated carrier lifetime estimated based on bulk trap-assisted SRH recombination model should be slightly exaggerated since it does not consider other non-radiation recombination such as Auger recombination and interface recombination. In any case, the carrier lifetime calculated by bulk trap-assisted SRH recombination model shows the similar trend at the same scale when compared with that obtained by TAS, suggesting that it is the intrinsic deep-level defect that restricts the carrier lifetime in Sb_2_S_3_.

## Discussion

For the Sb_2_S_3_ solar cell development, one of the major concerns is the efficiency improvement. The rational engineering of the defect properties benefits the carrier transport and suppressing the negative recombination, which further improve three device parameters, *V*_OC_, *J*_SC_, and FF. In particular, it has been acknowledged that there is a serious *V*_OC_ loss in the Sb_2_S_3_ solar cell. Fundamentally, the *V*_OC_ of solar cell originates from the split of quasi-Fermi level of electron and hole (Fig. 5c)^24,25^. The trap E2 and E3 in Sb-rich Sb_2_S_3_ films with large capture cross section and high trap density, especially trap energy level closer to Fermi level compared with H1 and H2 in S-rich Sb_2_S_3_. There is high possibility that the electron quasi-Fermi level is pinned near trap E2 and E3 in Sb-rich Sb_2_S_3_ owing to inefficient extraction of trapped photo-exited carriers. In contrast, S-rich Sb_2_S_3_ displays decrescent capture cross section, defects density and defects numbers which jointly give rise to suppressed recombination and prolonged carrier lifetime. This characteristic is able to alleviate the Fermi level pinning effects and improving *V*_OC_ ultimately.

An interesting finding in this study is that the existence of Sb_i_ in the Sb-rich Sb_2_S_3_ film generates less detrimental effect on the carrier lifetime, which should be related to Q1D crystal structure where the space between (Sb_4_S_6_)_n_ ribbons can afford impurities to certain degree. However, the S_i_ defect does not appear in the S-rich Sb_2_S_3_ film, it is most likely that the sulfur is easy to be evaporated out during the film deposition at high temperature. Finally, we find that the Sb-rich film displays two types of crucial defects, i.e., V_S_ and Sb_S_, while S-rich Sb_2_S_3_ film shows only one kind of critical defect, V_Sb_. Therefore, the S-rich Sb_2_S_3_ film seems more promising for achieving next efficiency breakthrough, provided that the V_Sb_ is well suppressed without introducing other deep-level defects.

## Methods

### Preparation of Sb_2_S_3_ thin films

The Sb_2_S_3_ films were deposited on FTO/CdS substrate preheated at 300 °C via thermal evaporation under pressure of 0.2–0.3 Pa. About 0.3 g Sb_2_S_3_ powder (99.9%, aladdin) was put into a tungsten boat equipped on the DC evaporator source, and the evaporating temperature was tuned through the DC current. As for co-evaporation, another 0.1 g S powder (99.999%, Sinopharm group) was needed to evaporate simultaneously with Sb_2_S_3_ powder. The films were deposited at a rate of 3–5 nm s^−1^. The final thickness of films was controlled around 200 nm via a film thickness gauge. Finally, the as-deposited films were post-annealed at 350 °C for 2 min on a preheated hot plate in a N_2_-filled glove box.

### Fabrication of Sb_2_S_3_ solar cells

The FTO glass (TEC-A7) was cleaned by DI water, isopropanol, acetone, and ethanol sequentially. Then FTO substrate was cleaned for 15 min by UV ozone prior to use as well. Next, the FTO substrate coated with 60 nm CdS film as ETL by using chemical bath deposition (CBD) method^4^. After treatment with CdCl_2_ (20 mg mL^−1^ methanol), then FTO/CdS substrate was heated at 400 °C for 10 min in open air. Subsequently, the Sb_2_S_3_ film was deposited on FTO/CdS substrate by thermal evaporation mentioned above. Spiro-OMeTAD (Advanced Election Technology Co., Ltd.) was utilized as HTL according our pervious report^5^. Finally, the Au back electrode was evaporated on the HTL under a pressure of 5 × 10^−4^ Pa. The active area was defined as 0.12 cm^−2^ by mask.

### Film characterizations

The morphologies of Sb_2_S_3_ thin films were characterized by Zeiss G450 SEM equipped with an EDS (Bruker) module. The crystal structure was measured by XRD (Bruker Advance D8 diffractometer) with Cu Kα radiation (*λ* = 1.5406 Å). Raman spectroscopy (Horiba JobinYvon, LabRAM HR800) was applied to analyze chemical bonds of the films with 532 nm laser excitation. XPS (Thermo Fisher) with a Monochrome Al Kα (1486.6 eV, 15 kV) was used to characterize the surface composition. The work function and valence band binding energy were measured by UPS (Thermo Escalab 250Xi, He I excitation 21.22 eV) which were recorded at 0 V samples bias in an ultrahigh vacuum chamber. Secondary ion mass spectroscopy was performed by TOF.SIMS (IONTOF, Germany). TAS of Sb_2_S_3_ films were measured by a Helios setup, where a nondegenerate pump–probe configuration was applied to probe the transient dynamics (50 fs to 7 ns). Additionally, the pump and probe laser pulses were generated by frequency doubling the fundamental output (Coherent Vitesse, 80 MHz, Ti-sapphire laser) and white light generated with a sapphire plate, respectively. The decay characteristics were fitted by biexponential model *y* = Σ*A*_i_exp(−*x*/*t*_i_), while carrier lifetime (*τ*) was obtained by *τ* = Σ*A*_i_*t*_i_^2^/Σ*A*_i_*t*_i_ (*i* = 2).

### Device characterizations

The *J–V* characteristics were performed by Keithley 2400 apparatus under solar-simulated AM 1.5 sunlight (100 mW cm^−2^) with a standard xenon-lamp-based solar simulator (Oriel Sol 3 A, Japan). Prior to test, the solar simulator illumination intensity was calibrated by a monocrystalline silicon reference cell (Oriel P/N 91150 V, with KG-5 visible color filter) which is also calibrated by the National Renewable Energy Laboratory (NREL). The EQE (model SPIEQ200) was measured using a single-source illumination system (halogen lamp) combined with a monochromator. The DLTS measurement was performed via a Phystech FT-1230 HERA-DLTS system equipped with a 10-mW red (635 nm wavelength) laser. The optical pulse was generated from the laser via systematical program control. The modified Boonton 7200 capacitance meter (1–75 kHz) was used to examine dynamic capacitance. An n–i–p structural device was selected as one sample, and the effective test area is about 0.01 cm^−2^. Then the samples were placed in a liquid-helium cryostat (Lakeshore 335,336). The DLTS temperature scan range was from 120 to 420 K at 2 K heating intervals. The pulse mode was set as electrical (pulse voltage) and optical (laser excitation) double pulse, and they were exerted and removed simultaneously. In detail, the reverse bias, pulse voltage, pulse width (electric and optical), and period width were −0.3 V, 0.1–0.5 V, 10 ms, and 100 ms, respectively.

### Reporting summary

Further information on experimental design is available in the Nature Research Reporting Summary linked to this paper.

## Supplementary information

Supplementary Information

Solar Cells Reporting Summary

## Data Availability

The source data that support the findings of this paper are available from the corresponding author on request. Source data are provided with this paper.

## Source data

Source Data
