# Supplementary material for: Revealing composition and structure dependent deep-level defect in antimony trisulfide photovoltaics
Source: Nat Commun. 2021 May 31;12:3260. doi: 10.1038/s41467-021-23592-0 (PMC8166839; doi:10.1038/s41467-021-23592-0)
Supplement: Supplementary file 1 — Supplementary Information [file 41467_2021_23592_MOESM1_ESM.pdf]

Supplementary Information

Revealing composition and structure dependent deep-level defect in antimony trisulfide photovoltaics

Weitao Lian[1,2] Chenhui Jiang[1,2], Yiwei Yin[1], Rongfeng Tang[1], Gang Li[1], Lijian Zhang[1], Bo Che[1] and Tao Chen[1,2]*

[1]Hefei National Laboratory for Physical Sciences at Microscale, CAS Key Laboratory of Materials for Energy Conversion, Department of Materials Science and Engineering, School of Chemistry and Materials Science, University of Science and Technology of China, Hefei, Anhui 230026, P. R. China

[2]Institute of Energy, Hefei Comprehensive National Science Center, Hefei, China

*E-mail: tchenmse@ustc.edu.cn

Supplementary Table 1 Characterisation of the element composition of Sb-rich and S-rich films deposited on soda-lime glass via energy dispersive X-ray spectroscopy (EDS).

**Films**	**Sb at%**	**S at%**	**S/Sb**	**S/Sb (average)**
	43.70	56.30	1.29	
	43.90	56.10	1.28	
Sb-rich	43.30	55.90	1.29	1.28
	43.80	56.20	1.28	
	44.10	55.90	1.27	
	38.40	61.60	1.60	
	39.80	60.20	1.51	
S-rich	38.20	61.80	1.62	1.55
	39.90	60.10	1.51	
	39.80	60.20	1.51	

Supplementary Table 2 Fitting results of TAS monitored at 545 nm wavelength.

**Films**	A_1	t_1**(ns)**	A_2	t_2**(ns)**	τ **(ns)**
Sb-rich	0.40	0.31	0.60	3.99	3.8
S-rich	0.09	0.38	0.91	18.69	18.7

Supplementary Table 3 Photovoltaic parameters of optimal Sb-rich and S-rich Sb_2S_3 devices.

**Devices**	V_{OC} **(V)**	J_{SC} **(mA.cm^{-2})**	***FF***	**η (%)**	R_s **(Ω)**	R_{sh} **(Ω)**
S-rich	0.74*	15.6	52.9	6.1	106.3	6184.6
	0.72	15.9*	54.3*	6.2*	100.4	6677.5
Sb-rich	0.68	15.4	47.6	5.0	116.5	1509.3

(* indicates the champion parameters.)

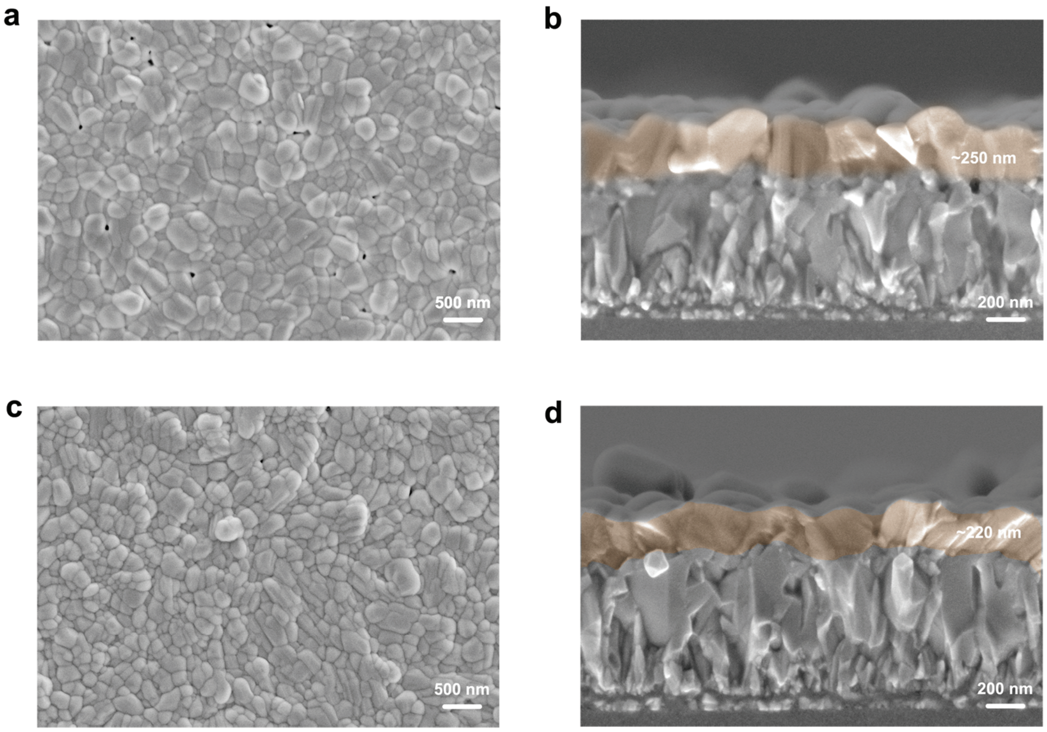

Supplementary Fig. 1 Films morphology characterizations. **a, b,** Surface and cross sectional morphology of Sb-rich Sb_2S_3 film. **c, d,** Surface and cross-sectional morphology of S-rich Sb_2S_3 film.

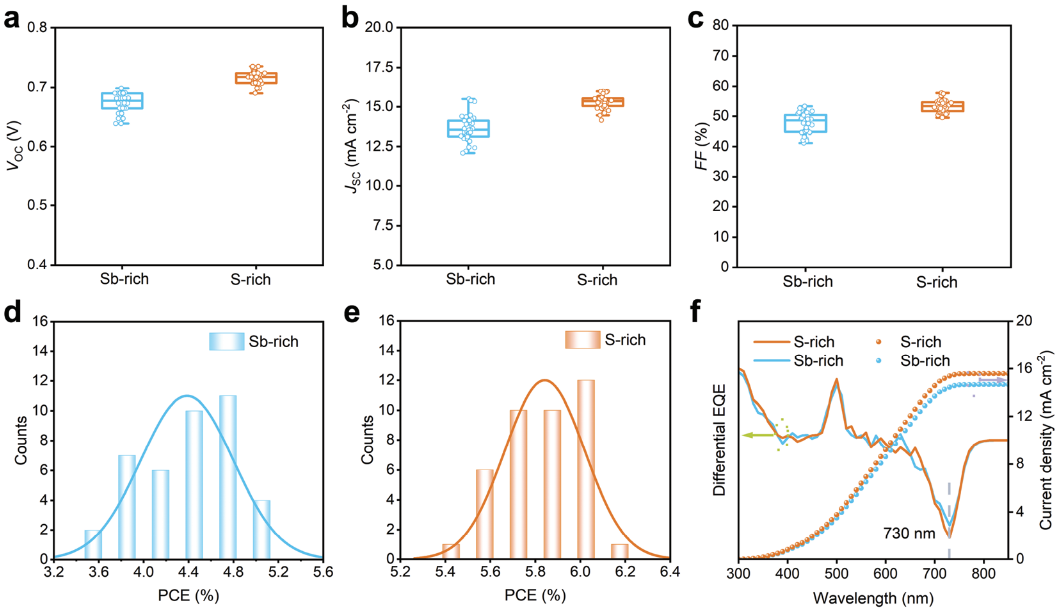

Supplementary Fig. 2 Device photovoltaic performance charaterizations. **a-c,** Statistical V_{OC}, J_{SC} and FF based on 40 individually fabricated Sb-rich and S-rich Sb_2S_3 devices. **d, e,** Statistical PCE distribution of 40 Sb-rich and S-rich Sb_2S_3 devices. **f** Differential EQE and integral current density of optimal Sb-rich and S-rich Sb_2S_3 based solar cells.

Supplementary note 1. Ultraviolet photoelectron spectroscopy.

To investigate the dependence of stoichiometry on the work function (Fermi level, E_F) and band structure of Sb_2S_3 films, we carried out ultraviolet photoelectron spectroscopy (UPS) characterizations. According to the secondary electron cutoff (SEC) and valence band (VB) position (Supplementary Fig. 3a and b), the E_F is calculated as -3.95 eV and -4.12 eV, respectively for Sb-rich and S-rich Sb_2S_3. Furthermore, we find that Sb-rich and S-rich Sb_2S_3 share an identical band gap of 1.70 eV corresponding to 730 nm absorption onset (Supplementary Fig. 2f). Hence, the CBM and VBM are determined to be -3.39 and -5.09 eV for S-rich Sb_2S_3. The CBM and VBM of Sb-rich Sb_2S_3 film

are -3.40 and -5.10 eV, respectively. Particularly, it is noted that the introduction of S has no impacts on the band structure and band gap but E_F solely. Regarding the downshift of E_F, we attribute it to some reduced shallow donor dopants accompanying with S addition[1]. Specially, it is worth noting that the S-rich Sb_2S_3 is still an n type semiconductor even if the E_F shift downward a little after S supplement.

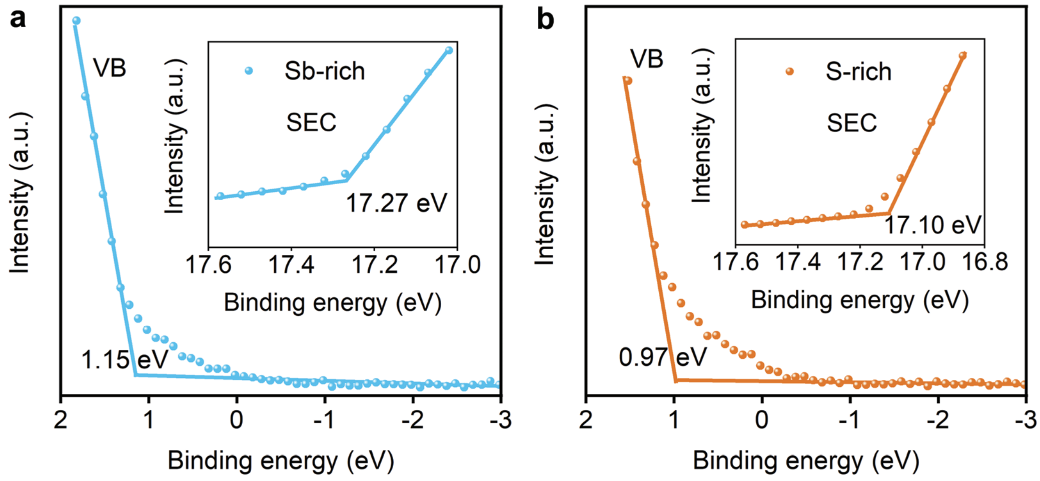

Supplementary Fig. 3 Band structure characterizations. **a, b,** Secondary electron (SEC) cutoff edge and valence band (VB) position of Sb-rich and S-rich Sb_2S_3 films.

Supplementary note 2. DLTS measurement background.

We conduct deep-level transient spectroscopy (DLTS) to detect the deep-level defects properties. The defect level is identified from DLTS signal using Fourier deconvolution algorithm, and Arrhenius plots obtained from defect peaks in DLTS signal are shown in Supplementary Fig. 4. The active energy (E_a, E_C-E_T or E_T-E_V) and capture cross section of electron traps and hole traps can be calculted by the Arrhenius equations (1) and (2)[2],

$$\ln(\tau_e \upsilon_{th,n} N_C) = \frac{E_C - E_T}{k_B T} - \ln(X_n \sigma_n), \quad (1)$$

$$\ln(\tau_e \upsilon_{th,p} N_V) = \frac{E_T - E_V}{k_B T} - \ln(X_p \sigma_p). \quad (2)$$

where τ_e ,N_C, N_V, E_C, E_T and E_V are emission time constant, conduction band state density, valence band state density, conduction band, trap energy level and valence band, respectively. $\upsilon_{th,n/p}$, $X_{n/p}$ and $\sigma_{n/p}$ represent thermal velocity, entropy factor and capture cross section for electron and hole, respectively. Hence, the E_a and σ can be extracted by the slope and y-axis intercept, separately. In addition, the trap density (N_T) could be obtained by equation (3)[2],

$$N_T = 2N_S \frac{\Delta C}{C_R}. \quad (3)$$

where N_S is the shallow donor concentration, C_R is the capacitance under reverse bias, while ΔC represents the amplitude of transient capacitance.

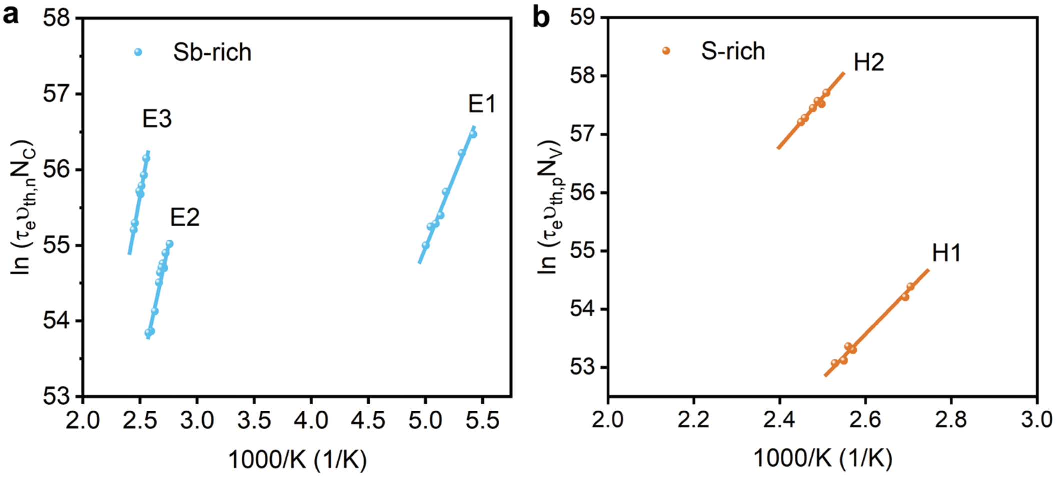

Supplementary Fig. 4 Arrhenius plots obtianed from DLTS for Sb-rich (**a**) and S-rich (**b**) Sb_2S_3.

Supplementary note 3. O_2 post-treatment.

To extensively study the influence of low-density oxygen, we adopted O_2 post-treatment for Sb_2S_3 film deposited by thermal evaporation. The as-deposited Sb_2S_3 film was firstly annealed in glovebox as the details described in Methods section (denote as-prepared), then it was placed into a tube furnace to heat at 200 °C for 20, 40 and 60 min in an O_2/Ar (v/v 5%) mixed atmosphere further with a flux of 60 sccm. The heating rate was set as 18 °C per minute, and sample was cooled down to ambient temperature naturally after O_2 post-treatment.

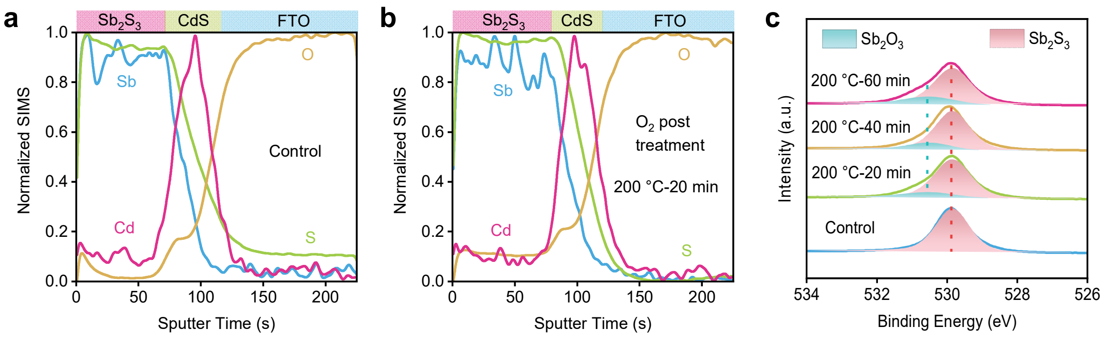

Supplementary Fig. 5 Secondary ion mass spectroscopy (SIMS) and XPS spectra. **a, b,** SIMS of the as-prepared Sb-rich Sb_2S_3 sample (Control) and annealed in O_2/Ar (v/v 5%) at 200 °C for 20 min, respectively. **c** XPS spectra of the as-prepared Sb-rich Sb_2S_3 sample and annealed in O_2/Ar (v/v 5%) at 200 °C for 20-60 min.

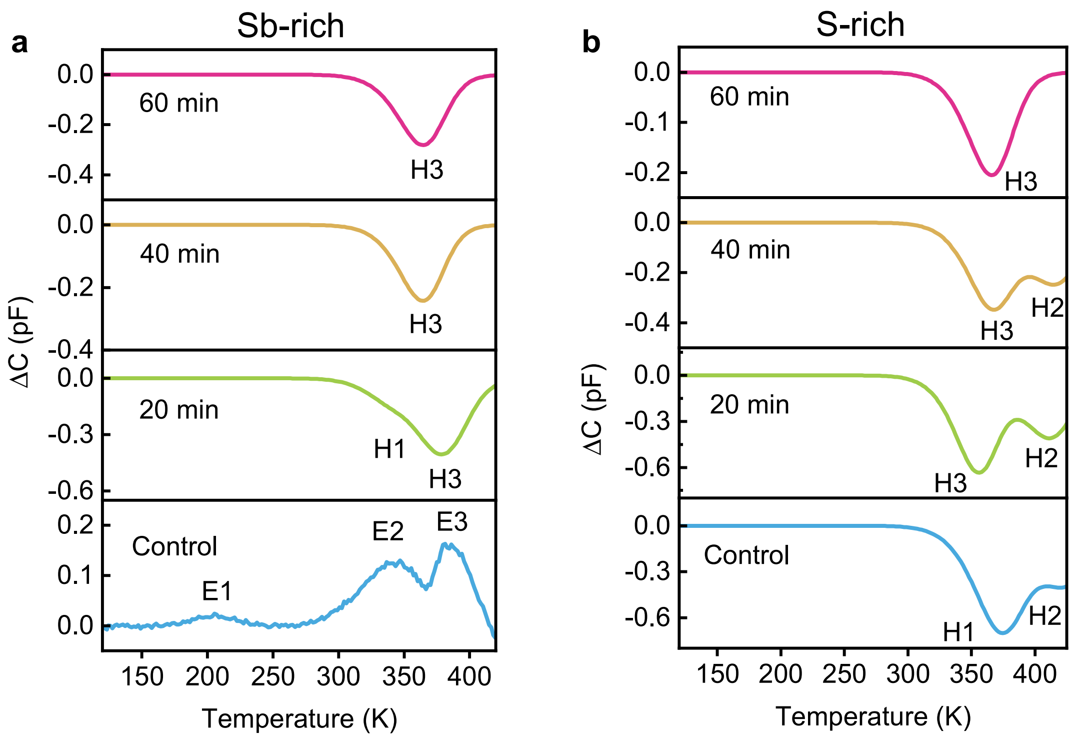

Supplementary Fig. 6 DLTS characterizations. **a,** DLTS characterizations of as-prepared Sb-rich Sb_2S_3 film (denoted as Control) and annealed in O_2/Ar (v/v 5%) at 200 °C for 20-60 min. **b,** DLTS characterizations of as-prepared S-rich Sb_2S_3 film (Control) and annealed in O_2/Ar (v/v 5%) at 200 °C for 20-60 min. The reverse bias, pulse voltage, double pulse (electric & optical) width and period width of DLTS were set as -0.3 V, 0.5 V, 10 ms and 100 ms, respectively.

Supplementary Table 4 The E_T, σ and N_T and N_S extracted from DLTS measurement of as-prepared Sb-rich Sb_2S_3 film (denoted as Control) and annealed in O_2/Ar (v/v 5%) at 200 °C for 20-60 min.

**Sb-rich Sb_2S_3**		**Trap**	**E_T (eV)**	**σ (cm^2)**	**N_T (cm^{-3})**	**N_S (cm^{-3})**
**Control**		E1	E_C-0.291	2.19×10^{-17}	6.07×10^{13}	5.00×10^{16}
		E2	E_C-0.621	6.10×10^{-16}	4.24×10^{14}	5.00×10^{16}
		E3	E_C-0.709	2.00×10^{-15}	6.79×10^{14}	5.00×10^{16}
**O_2 post-treatment**	20 min	H1	E_V+0.612	3.14×10^{-16}	2.86×10^{14}	2.94×10^{16}
		H3	E_V+0.674	1.20×10^{-16}	8.32×10^{14}	2.94×10^{16}
	40 min	H3	E_V+0.682	8.45×10^{-16}	1.53×10^{14}	2.63×10^{16}
	60 min	H3	E_V+0.661	2.37×10^{-16}	3.29×10^{14}	3.58×10^{16}

Supplementary Table 5 The E_T, σ and N_T and N_S extracted from DLTS measurement of as-prepared S-rich Sb_2S_3 film (denoted as Control) and annealed in O_2/Ar (v/v 5%) at 200 °C for 20-60 min.

**S-rich Sb_2S_3**		**Trap**	**E_T (eV)**	**σ (cm^2)**	**N_T (cm^{-3})**	**N_S (cm^{-3})**
**Control**		H1	E_V+0.625	4.60×10^{-17}	1.42×10^{15}	4.54×10^{16}
		H2	E_{V+}0.759	1.40×10^{-16}	8.32×10^{14}	4.54×10^{16}
**O_2 post-treatment**	20 min	H2	E_V+0.753	2.23×10^{-16}	5.88×10^{14}	2.67×10^{16}
		H3	E_V+0.661	4.41×10^{-16}	8.72×10^{14}	2.67×10^{16}
	40 min	H2	E_V+0.730	9.22×10^{-17}	2.54×10^{14}	2.15×10^{16}
		H3	E_V+0.669	2.86×10^{-16}	3.33×10^{14}	2.15×10^{16}
	60 min	H3	E_V+0.668	7.70×10^{-16}	3.43×10^{14}	4.62×10^{16}

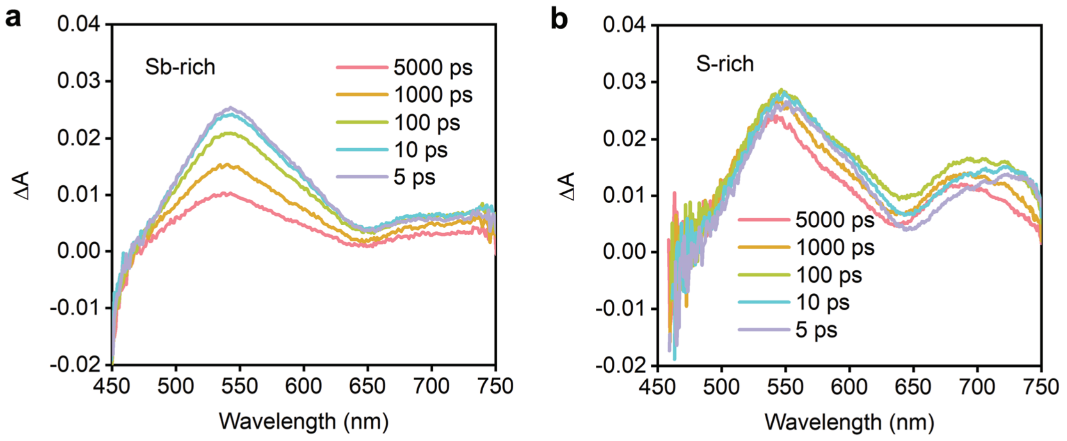

Supplementary Fig. 7 Carrier transport kinetics. **a, b,** TAS for Sb-rich and S-rich Sb_2S_3 films on soda-lime glass tracked at 5, 10, 100, 1000 and 5000 ps after pulsed excitation at 400 nm.

Supplementary reference.

1. Yin, Y. et al. Composition engineering of Sb_2S_3 film enabling high performance solar cells. *Sci. Bull.* **64**, 136-141 (2019).

2. Lang, D. Deep-level transient spectroscopy: A new method to characterize traps in semiconductors. *J. Appl. Phys.* **45**, 3023-3032 (1974).
