# Supplementary material for: Revealing composition and structure dependent deep-level defect in antimony trisulfide photovoltaics
Source: Nat Commun. 2021 May 31;12:3260. doi: 10.1038/s41467-021-23592-0 (PMC8166839; doi:10.1038/s41467-021-23592-0)
Supplement: Supplementary file 2 — Solar Cells Reporting Summary [file 41467_2021_23592_MOESM2_ESM.pdf]

nature research

Corresponding author(s): Tao Chen

Solar Cells Reporting Summary

Nature Research wishes to improve the reproducibility of the work that we publish. This form is intended for publication with all accepted papers reporting the characterization of photovoltaic devices and provides structure for consistency and transparency in reporting. Some list items might not apply to an individual manuscript, but all fields must be completed for clarity.

For further information on Nature Research policies, including our data availability policy, see Authors & Referees.

Experimental design

Please check: are the following details reported in the manuscript?

1. Dimensions

Item	Yes/No	Details
Area of the tested solar cells	☒ Yes ☐ No	The area of tested solar cells is 0.12 cm2 (described in Methods, fabrication of Sb2S3 solar cells)
Method used to determine the device area	☒ Yes ☐ No	The active area was determined by the mask

2. Current-voltage characterization

Item	Yes/No	Details
Current density-voltage (J-V) plots in both forward and backward direction	☐ Yes ☒ No	No current density-voltage plots in forward direction, since it is not the subject of the article
Voltage scan conditions<br>*For instance: scan direction, speed, dwell times*	☒ Yes ☐ No	J-V curves were measured by reverse scan (0.75 V to -0.1 V) with a scanning rate of 32mV/s (voltage step of 10 mV) (Figure 3c, and Device Characterizations)
Test environment<br>*For instance: characterization temperature, in air or in glove box*	☒ Yes ☐ No	Performance measurements were tested in air ambient environments under room temperature (Methods)
Protocol for preconditioning of the device before its characterization	☐ Yes ☒ No	No preconditioning was used before its characterization
Stability of the J-V characteristic<br>*Verified with time evolution of the maximum power point or with the photocurrent at maximum power point; see ref. 7 for details.*	☐ Yes ☒ No	The stability of the J-V characteristics is not shown in this manuscript.

3. Hysteresis or any other unusual behaviour

Item	Yes/No	Details
Description of the unusual behaviour observed during the characterization	☐ Yes ☒ No	No measurements of hysteresis behaviour in this report, since it is not the subject of the article.
Related experimental data	☐ Yes ☒ No	No related experimental data in this manuscripts.

4. Efficiency

Item	Yes/No	Details
External quantum efficiency (EQE) or incident photons to current efficiency (IPCE)	☒ Yes ☐ No	The EQE was shown in Figure 3d
A comparison between the integrated response under the standard reference spectrum and the response measure under the simulator	☒ Yes ☐ No	The integrated Jsc from EQE spectra agree well with the Jsc from J-V measurements (Figure 3c and Supplementary Figure 2f)
For tandem solar cells, the bias illumination and bias voltage used for each subcell	☐ Yes ☒ No	No tandem cells reported in this manuscript

5. Calibration

Item	Yes/No	Details
Light source and reference cell or sensor used for the characterization	☒ Yes ☐ No	A standard xenon-lamp-based solar simulator (Oriel Sol 3A, Japan) is used for the measurements (Methods, Devices Characterizations)
Confirmation that the reference cell was calibrated and certified	☒ Yes ☐ No	The solar simulator illumination intensity was calibrated by a monocrystalline silicon reference cell (Oriel P/N 91150 V, with KG-5 visible color filter) calibrated by the National Renewable Energy Laboratory (NREL) (Methods, Devices Characterizations)

Calculation of spectral mismatch between the reference cell and the devices under test	☒ Yes ☐ No	Mismatch factor of 1 was used in our measurements

6. Mask/aperture

Size of the mask/aperture used during testing	☒ Yes ☐ No	The size of the mask is 0.12 cm2 (Methods)
Variation of the measured short-circuit current density with the mask/aperture area	☐ Yes ☒ No	No measurement of the device with different the mask/aperture area was conducted

7. Performance certification

Identity of the independent certification laboratory that confirmed the photovoltaic performance	☐ Yes ☒ No	No performace certification was reported in this manuscript
A copy of any certificate(s)<br>*Provide in Supplementary Information*	☐ Yes ☒ No	No copys of any cerficates in this manuscript

8. Statistics

Number of solar cells tested	☒ Yes ☐ No	40 independently fabricated devices were tested (Supplementary Figure 2a-e)
Statistical analysis of the device performance	☒ Yes ☐ No	The statistical analysis of device performance were shown in Supplementary Figure 2a-e

9. Long-term stability analysis

Type of analysis, bias conditions and environmental conditions<br>*For instance: illumination type, temperature, atmosphere humidity, encapsulation method, preconditioning temperature*	☐ Yes ☒ No	No long-term stability measurement reported in this manuscript, since the little relevance to the subject.
